# The Pharmaceutical Association of Georgia

**Published:** 1882-04-20

**Authors:** 


THE PHARMACEUTICAL ASSOSIATION of Georgia met in Atlanta on 11th inst. The officers elected for the ensuing year are: J. W. Rankin, President; A. Solomons, of Savannah, 1st Vice-President; G. J. Howard, of Atlanta, 2d Vice-President; A. M. Brannon, of Columbus, 3d Vice-President. Secretary, J. A. Scrupline, of Savannah. J. L. Massenburg, of Macon, Treasurer.

The next meeting of the Association will be in Athens, on 2nd Monday of April 1882.

